# Cilium-by-cilium: unveiling hidden proteomic diversity and the molecular basis of ciliopathies

**DOI:** 10.1038/s41392-025-02555-7

**Published:** 2026-01-10

**Authors:** Mario Chiong, Hongliang Li, Sergio Lavandero

**Affiliations:** 1 Advanced Center for Chronic Diseases (ACCDiS), Facultad de Ciencias Químicas y Farmacéuticas & Facultad de Medicina, Universidad de Chile, Santiago, Chile; 2 Departamento de Bioquímica y Biología Molecular, Facultad de Ciencias Químicas y Farmacéuticas, Universidad de Chile, Santiago, Chile; 3 Gannan Innovation and Translational Medicine Research Institute, Gannan Medical University, Ganzhou, Jiangxi China; 4 Department of Cardiology, Renmin Hospital of Wuhan University, Wuhan, China; 5 Medical Science Research Center, Zhongnan Hospital of Wuhan University, Wuhan, China; 6 Grupo de Biología y Genética, Instituto de Ciencias Biomédicas (ICBM), Facultad de Medicina, Universidad de Chile, Santiago, Chile; 7 Department of Internal Medicine (Cardiology Division), University of Texas Southwestern Medical Center, Dallas, TX USA

**Keywords:** Cell biology, Structural biology, Metabolic disorders

In a recent study published in Cell, Hansen et al. applied antibody-based spatial proteomics and advanced confocal imaging to build a comprehensive single-organelle-resolution proteome atlas of the human primary cilium from three cell types —hTERT-RPE1 cells from the embryonic retina, RPTEC/TERT1 cells from the epithelial kidney proximal tubules, and the mesenchymal, multipotent, stromal ASC52telo cells—, identifying 715 proteins and revealing marked cell-type and single-cilium variability.^1^ This work demonstrates that primary cilia are highly customizable signaling hubs whose proteomic diversity provides a robust framework for understanding cilia-dependent signaling and uncovering the molecular basis of ciliopathies.

**Fig. 1:**
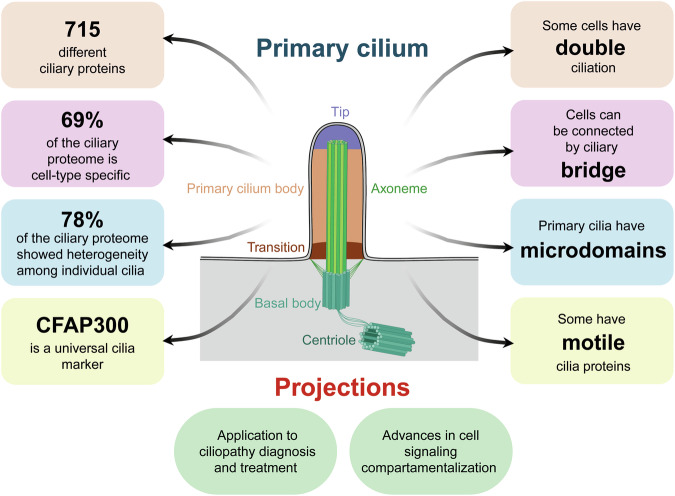
Characteristics of the primary cilia. The primary cilium is a specialized organelle that senses external signals and transmits them to the cell body. It originates from the mother centriole, which transforms into the basal body and serves as the base for axoneme formation. The primary cilium features four distinct regions: the tip, the ciliary body, the transition zone, and the basal body. The left rounded boxes illustrate the main characteristics of the primary cilia proteome, while the right rounded boxes highlight key structural features of the cilium. Ellipses represent insights gained from the analysis of the ciliary proteome

Primary cilia are antenna-like structures present on nearly every vertebrate cell type and play a critical role in cellular sensing and signal transduction, which are essential for development and tissue homeostasis. They are deeply involved in sensing mechanical and chemical cues and in regulating development, cell growth, and organogenesis.^2^ Albeit the previous description that cells have a single immotile primary cilium,^3^ Hansen et al. described the presence of cells with two adjacent cilia in hTERT-RPE1 and RPTEC/TERT1 cells, the presence of potential ciliary bridges between RPTEC/TERT1 cells, and the presence of proteins believed to be specific for motile cilia or sperm flagella in RPTEC/TERT1 cilia (Fig. 1).

The antibody-based approach used by Hansen et al. provided significant advantages over previous proximity-labeling methods like APEX and BioID,^4^ which are limited by bulk population averaging, off-target labeling, fusion tag artifacts, and their inability to resolve single-cilium diversity or to be applied in patient tissue samples. Hansen et al. manually annotated nearly 20,000 confocal images, assigning protein localization to distinct cilium-specific regions: basal body, transition zone, primary cilium body, and tip. This enabled a nuanced spatial map of each protein’s location within the organelle.

Proteomic data revealed that the primary cilium’s protein composition is far more variable than any other organelle, including mitochondria or the ER. Of the 715 identified proteins, 91 had never been linked to cilia before; 69% were present in only one or two of the three cell types studied, suggesting extensive customization by tissue context. For example, the classical ciliary protein ADCY3 showed differing localization in adipose, kidney, and retinal cell cilia, while PI3K was ciliary only in some cell types. This level of customization outweighs that seen for any other measured cellular compartment. Even within a single cell type, ciliary composition varied considerably: 78% of proteins showed different spatial distributions or abundances at the single-cilium level. Proteins that are involved in basic ciliary structure, like tubulin modifiers, tended to remain relatively stable, but those engaged in signaling, development, or differentiation varied greatly. Key signaling proteins, including GPCRs such as LPAR3, SSTR3, and HTR7, showed marked abundance variation across cilia, even within highly synchronized cell populations. These findings indicate that protein diversity reflects biologically regulated states rather than random fluctuations or technical artifacts. However, it will be essential to determine to what extent these differences reflect stable cell-state diversification, stochastic fluctuations, local post-translational regulation, or dynamic remodeling in response to microenvironmental cues, and how such diversity tunes cilia-dependent signaling outputs in specific tissues.

Functional annotation of the ciliary proteome revealed an unexpected depth and complexity in signaling machinery. Protein kinases such as AKT1, AKT3, and CAMK1G; components of major pathways such as PDGF, TGF-β, and Wnt; and dozens of GPCRs were found in the cilium, far expanding the known list of ciliary signaling players. Notably, most ciliary proteins also localized to other cellular compartments (99%), suggesting extensive crosstalk between cilia and the greater signaling cellular network. This principle extended to metabolic enzymes, ubiquitin ligases, and transcription factors such as CREB1 and ATF3, suggesting that the cilium acts as both a metabolic and regulatory hub, not just a signal receiver. However, their precise contribution to the signaling machinery under physiological conditions in vivo has not been demonstrated. This uncertainty is exemplified by canonical Wnt signaling, which appears grossly intact in mouse embryos and fibroblasts lacking cilia despite the presence of multiple Wnt-related proteins in the organelle.^5^ The study also mapped functionally distinct ciliary subdomains, revealing at least 20 protein clusters per cell line, each associated with unique molecular activities. For example, transition zone clusters were linked to protein trafficking, while ciliary tip clusters were enriched in kinases involved in autophosphorylation. Such compartmentalization likely supports simultaneous, selective signaling processes while preventing interference between incompatible pathways. This spatial functional diversity points to a new organizational principle in ciliary biology.

Of all 715 proteins, only ARL13B and CFAP300 labeled cilia exclusively and stably across all cell types, with CFAP300 emerging as a universal marker. CFAP300 reliably and specifically stains primary cilia in many tissues, including the kidney, liver, and thyroid, without non-ciliary cross-reactivity. This advance will facilitate ongoing basic and clinical studies in cilia biology and aid in the diagnosis of ciliopathies.

Dysfunction of primary cilia leads to a broad spectrum of disorders known as ciliopathies. Hansen et al. reported that 324 of the mapped ciliary genes have known human disease associations; 338 are OMIM-reported but lack phenotype links; and 53 are entirely novel. Among these, 14 newly discovered ciliary proteins are strongly implicated in neurodevelopment, including NRCAM and DDC, suggesting new avenues for research into brain function and neurodevelopmental disorders. Most compellingly, a child with multiple ciliopathy-like symptoms was found to carry a novel CREB3 mutation. Therefore, the atlas offers a new approach to identifying candidate disease genes in undiagnosed patients with multisystem disorders associated with ciliopathies.

Despite its breadth and single-organelle resolution, this atlas remains an in vitro, antibody-based snapshot of the ciliary proteome in a limited set of human cell lines and thus does not directly establish causal or physiological roles for most detected proteins in vivo. Many of the signaling components mapped to primary cilia have not yet been functionally validated in animal models, underscoring that localization alone does not prove pathway dependence on the organelle. Future work integrating this spatial proteomics framework with genetic perturbations, live-cell imaging, and spatial transcriptomics or metabolomics in organoids and animal models will be essential to link ciliary localization to signaling output, tissue-specific physiology, and disease phenotypes. Moreover, systematic application of this approach to patient-derived cells and well-characterized ciliopathy cohorts could help prioritize variant interpretation and uncover how organ-level context modulates ciliary signaling networks in vivo.

Overall, the generation of a comprehensive, spatially resolved human primary cilium protein atlas sets a new standard for understanding ciliary function and its role in health and disease. The work fundamentally reconceptualizes the cilium as a customizable signaling center and a resource for unraveling mechanisms of genetic disease, even though the functional and physiological relevance of many ciliary-localized proteins remains to be established experimentally.
